# A Five-Parameter Logistic Model to Predict the Possibility of Misdiagnosis for Improving the Specificity of Lugol Chromoendoscopy in the Diagnosis of Esophageal Neoplastic Lesions

**DOI:** 10.3389/fonc.2021.763375

**Published:** 2022-01-03

**Authors:** Zijun Guo, Lingjun Meng, Shuxin Tian, Lan Chen, Huiying Shi, Mengke Fan, Rong Lin

**Affiliations:** ^1^ Department of Gastroenterology, Union Hospital, Tongji Medical College, Huazhong University of Science and Technology, Wuhan, China; ^2^ Department of Gastroenterology, The First Affiliated Hospital, Shihezi University, Shihezi, China; ^3^ Department of Gastroenterology, The Sixth Hospital, Jianghan University, Wuhan, China

**Keywords:** esophageal neoplasia, esophageal squamous cell carcinoma, Lugol chromoendoscopy, misdiagnosis, risk factors

## Abstract

**Background:**

Lugol chromoendoscopy (LCE) is a technique that is inexpensive and convenient for screening esophageal neoplastic lesions. However, the specificity of LCE is limited. The purpose of this study was to determine the risk characteristics of lesions related to false-positive results for LCE.

**Methods:**

In this retrospective study, 871 lesions in 773 patients scheduled for LCE in Wuhan Union Hospital and First Affiliated Hospital of Shihezi University between April 2013 and October 2018 were enrolled. The 871 lesions were used to determine the diagnostic performance of LCE for detecting esophageal neoplastic lesions and were divided into an LCE-positive group (627 lesions) and an LCE-negative group (244 lesions). Six hundred and twenty-seven unstained/understained lesions from 563 patients were used to determine the significant risk factors for misdiagnosis of neoplasms by LCE. Among them, 358 lesions and 269 lesions were classified into the misdiagnosed group and correctly diagnosed group, respectively. A multivariate logistic regression analysis was conducted for suspected esophageal neoplastic lesions during the LCE examination.

**Results:**

The sensitivity, specificity, and overall accuracy for LCE were 100%, 40.5%, and 58.9%, respectively. Among 13 characteristics of lesions, lesions with branching vascular network (OR 4.53, 95% CI 2.23–9.21, *p* < 0.001), smooth lesions (OR 2.40, 95% CI 1.38–4.18, *p* = 0.002) under white light endoscopy (WLE), lesions with a size < 5 mm (OR 3.06, 95% CI 1.38–6.78, *p* = 0.006), ill-demarcated lesions (OR 7.83, 95% CI 4.59–13.37, *p* < 0.001), and pink color sign (PCS)-negative (OR 4.04, 95% CI 2.38–6.84, *p* < 0.001) lesions after reaction with iodine solution were independent risk factors for misdiagnosis as neoplastic lesions by LCE.

**Conclusion:**

LCE has a high sensitivity but limited specificity for screening esophageal neoplastic lesions. For unstained or understained lesions, branching vascular network or smooth appearance under WLE, a size < 5 mm in diameter, ill-demarcated, or PCS-negative lesions after staining are related to the misdiagnosis of esophageal neoplastic lesions by LCE based on logistic regression. The multivariate logistic model may be used to predict the possibility of misdiagnosis and help improve the specificity of LCE in diagnosing esophageal neoplastic lesions.

## Introduction

Esophageal squamous cell cancer (ESCC) is one of the most common malignant tumors, ranking seventh in the world (1). The prognosis is poor, and the mortality is high for ESCC tumors detected at a late stage. The best results are achieved with early diagnosis (2, 3). However, most ESCC cases present in late stages, resulting in delayed diagnosis of the disease. If the disease is detected in the early stages, the overall 5-year survival rates, which are approximately 15%–25%, will be considerably improved (4). It has been reported that the 5-year survival rates of ESCC at stages 0, I, and IIA–IVB were 83%, 47%, and 0%, respectively (5). Thus, screening esophageal squamous cell neoplastic lesions at an early stage is crucial to improve the prognosis of ESCC.

As early neoplastic changes cannot be readily identified by white light imaging (WLI), the current use of conventional endoscopy for screening neoplastic lesions has limitations. Lugol chromoendoscopy (LCE) has remained the primary technique for detecting and screening ESCC (6, 7), especially in China. In addition, narrow-band imaging (NBI), another optical image-enhanced technology that can improve the visualization of microvascular structure and mucosal pattern (8, 9), exhibits high specificity and accuracy in the diagnosis of esophageal neoplastic lesions with a time-saving advantage (10). However, NBI endoscopy requires expensive devices and experienced endoscopists. Ishihara et al. reported that the diagnostic accuracy of NBI in experienced endoscopists was 11%–15% higher than that of inexperienced endoscopists (11). Consequently, its widespread use is limited in some places due to various difficulties.

The LCE technique, which is not only cheap but also easy to perform, can be easily mastered by endoscopists. Consequently, LCE has been utilized as the preferred method of screening esophageal neoplastic lesions in some areas (12). However, LCE presents high sensitivity (91%–100%) (13, 14) but low specificity (40%–95%) for the detection of early ESCC lesions (15, 16), leading to a high false-positive rate and the need for unnecessary biopsies. Some benign lesions, including esophagitis, ectopic mucosa, atrophy, and epithelial keratinization, can also appear unstained or understained under LCE (17).

As alluded to above, it is urgent and crucial to improve the specificity of LCE to promote its application in ESCC screening. The primary endpoint of this study was to determine the risk factors related to poor specificity and to improve the diagnostic efficiency of LCE in esophageal neoplastic lesions.

## Materials and Methods

### Study Design and Patients

In this retrospective double-center study, 773 patients who were scheduled for LCE in Wuhan Union Hospital and First Affiliated Hospital of Shihezi University between April 2013 and October 2018 were enrolled and analyzed based on endoscopic images. A total of 871 lesions in these 773 patients were used to determine the diagnostic performance of LCE for detecting esophageal neoplastic lesions. Patients were divided into an LCE-positive group (627 lesions) and an LCE-negative group (244 lesions).

The exclusion criteria were as follows (1): lesions were not confirmed by histopathology or could not be adequately assessed by histopathology (2); lesions were pathologically diagnosed as esophageal adenocarcinoma or Barrett’s esophagus (3); lesions were diagnosed as ulcers by naked-eye observation or pathological evaluation (esophageal squamous epithelium may disappear and result in direct exposure of the muscular layer in this condition) (4); endoscopic images of lesions with poor quality; and (5) patients accepted radiotherapy or chemoradiotherapy before endoscopy examination.

In addition, 563 patients with 627 unstained/understained lesions were used to determine the significant risk factors for misdiagnosis in LCE. These patients were divided into two groups (1): Misdiagnosed group (Group A), lesions diagnosed as neoplastic lesions by LCE but eventually pathologically diagnosed as nonneoplastic lesions (2); Correctly diagnosed group (Group B), lesions diagnosed as neoplastic lesions by LCE and finally confirmed by pathological examination.

All data were collected from the database of the Endoscopy Center in both Wuhan Union Hospital and First Affiliated Hospital of Shihezi University.

### Endoscopic Examination and Grading of Staining Patterns

**Figure 1:**
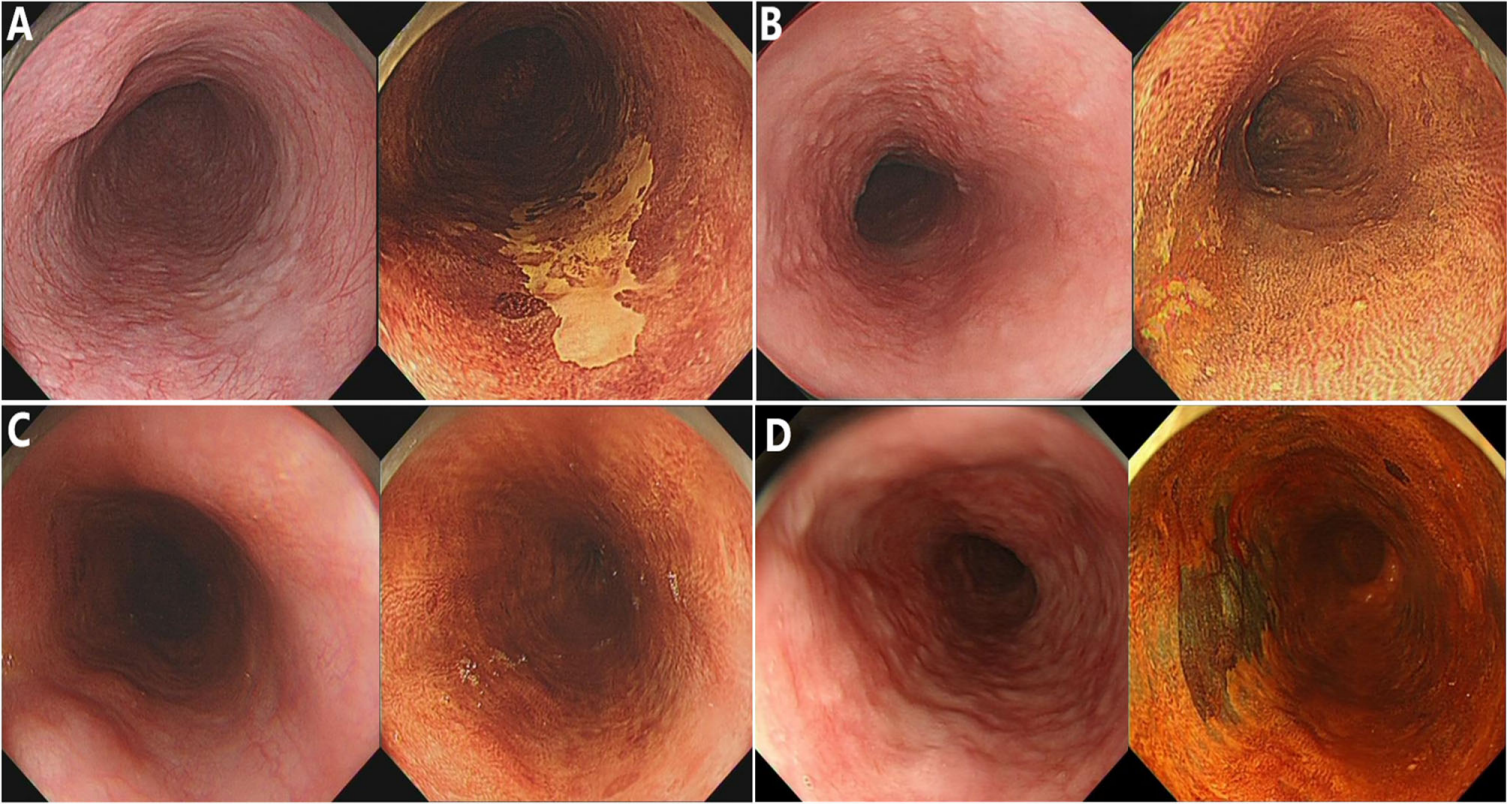
The staining patterns of Lugol chromoendoscopy (LCE) were graded and classified into four types. **(A)** Unstained; **(B)** understained, less intense staining; **(C)** normal greenish-brown staining; **(D)** hyperstaining.

In the current study, endoscopic procedures were performed according to a standard protocol using a conventional endoscope (GIF-HQ290; Olympus Optical, Tokyo, Japan). LCE of the esophageal mucosa was performed using the Lugol dye-staining method. A 1.0% solution of Lugol dye was used in this study. The grading of staining patterns was divided into four groups (18): hyperstaining (grade I); normal greenish-brown staining (grade II); understained (grade III); and unstained (grade IV) (**Figure 1**). Grades I–II were considered benign and defined as LCE negative, whereas grades III–IV were considered neoplastic lesions and defined as LCE positive. All endoscopic procedures were performed by experienced endoscopic physicians who had performed greater than 10,000 gastroscopies.

### Biopsy Specimens and Histopathology

**Figure 2:**
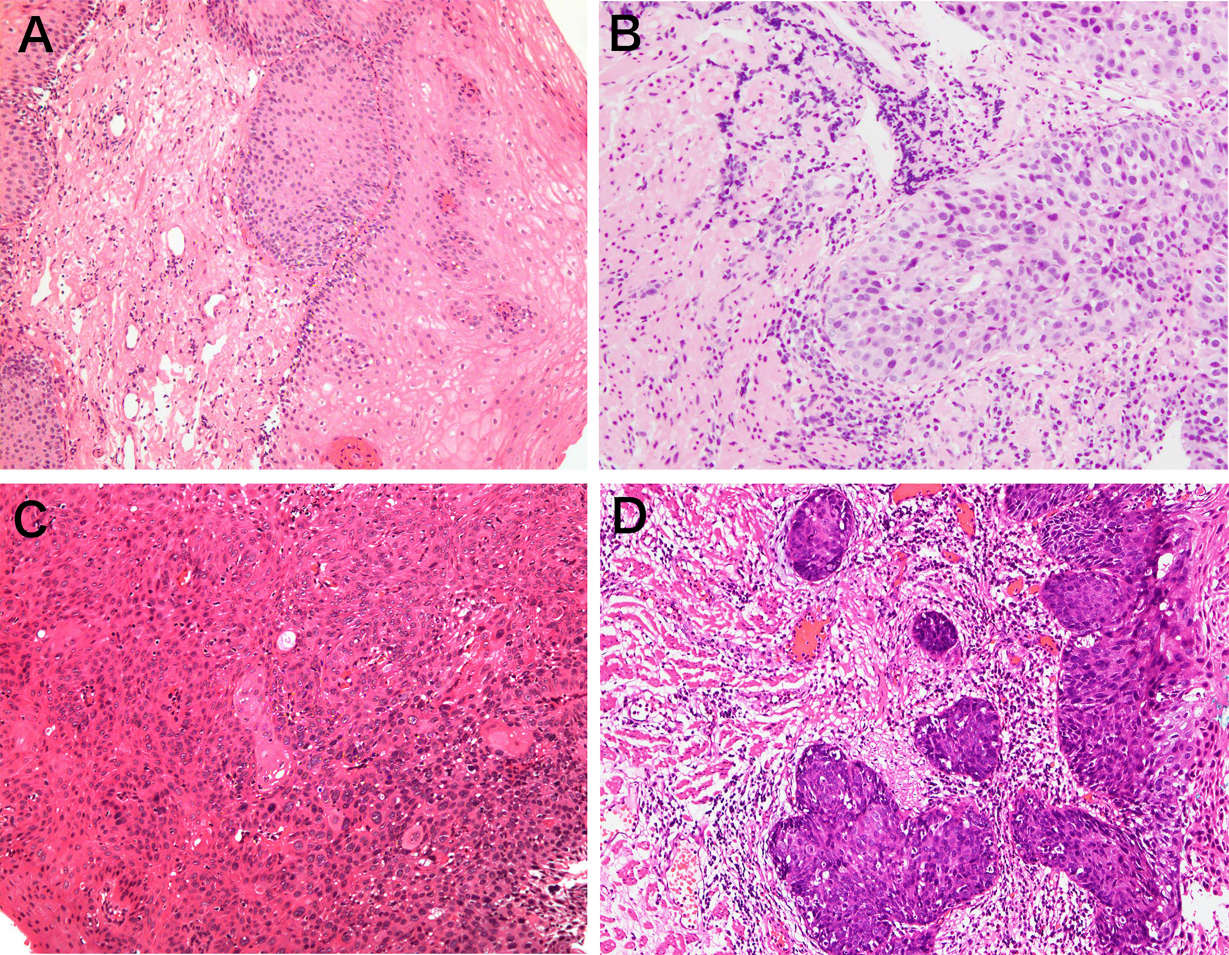
Representative pictures of pathological classification. **(A)** Negative for neoplasia/dysplasia (including normal, reactive, regenerative, hyperplastic, atrophic, and metaplastic epithelium); **(B)** noninvasive low-grade neoplasia; **(C)** noninvasive high-grade neoplasia; **(D)** invasive neoplasia.

Biopsy specimens were obtained from all enrolled lesions in the current study. All biopsy specimens underwent standard histologic assessment by experienced pathologists who were blinded to the clinical characteristics of all patients. Pathological diagnosis was made according to the World Health Organization classification (19) (1): negative for neoplasia/dysplasia (including normal, reactive, regenerative, hyperplastic, atrophic, and metaplastic epithelium) (2); noninvasive low-grade neoplasia (3); noninvasive high-grade neoplasia; and (4) invasive neoplasia (**Figure 2**). The histopathologic diagnosis served as the criterion standard.

### Imaging Evaluation

**Figure 3:**
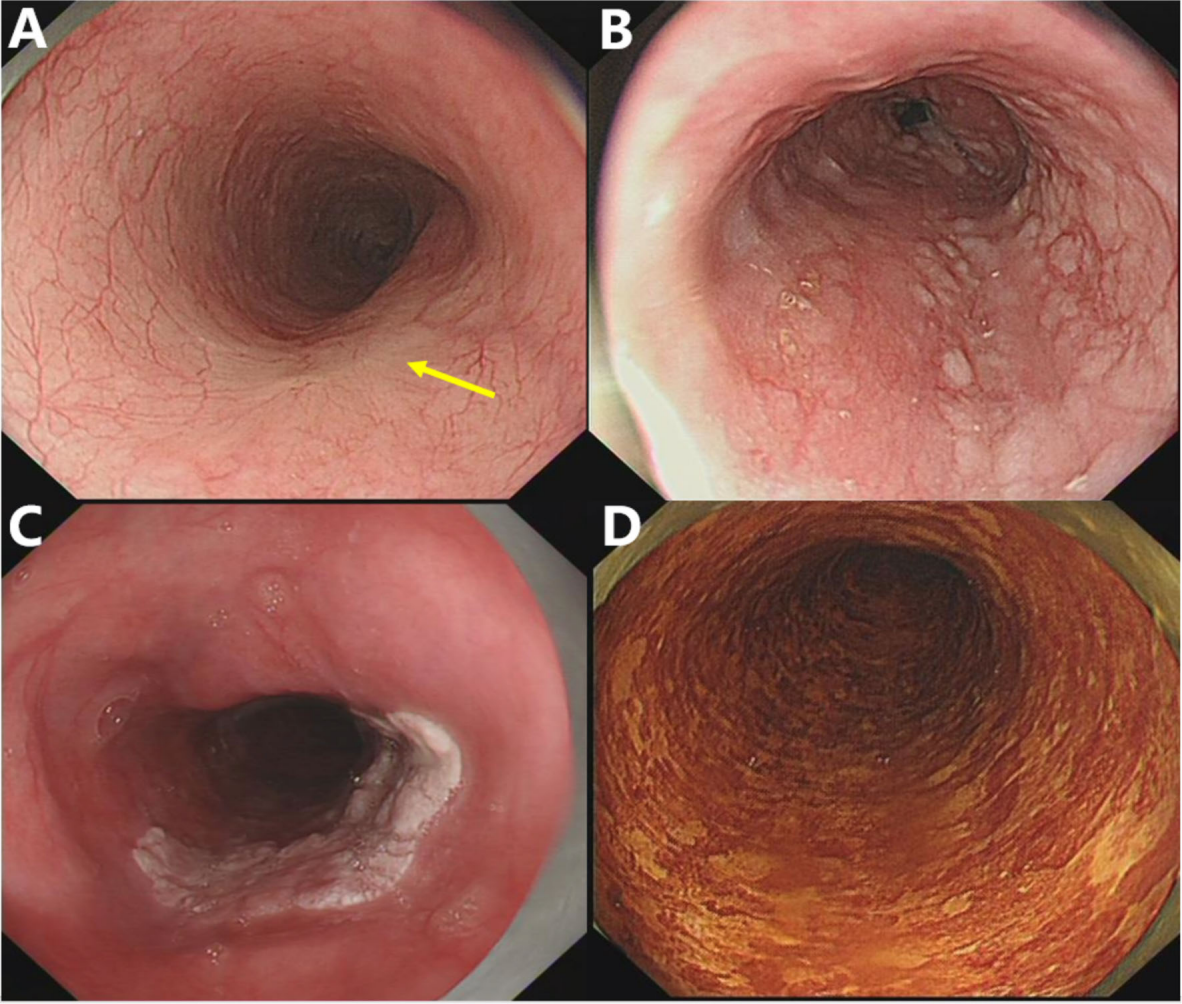
Representative pictures of specific endoscopic characteristics. **(A)** Disorder, disappearance, or truncation of the vascular network; **(B)** nodule, lesions with a diameter of less than 1 cm, bulging surface, and rough/erosive mucosa; **(C)** plaque, massive lesions that are mostly white and slightly raised from the mucosal surface with clear borders; **(D)** speckled esophagus, multiple Lugol-unstained speckles were present throughout the esophagus.

To adjust for evaluation bias during image analysis, two endoscopists who were blinded to the histologic results reviewed all endoscopic images of the included patients. When there are different opinions, the final result will be decided by discussion. The endoscopic images of each subject were evaluated based on the following aspects (1): macroscopic appearance, number of lesions, location, vascular network, and hyperemia under WLE (2); size, margin, shape, rugosity, and pink color sign (PCS) under LCE; and (3) other endoscopic characteristics, including erosion, nodule, and plaque (**Figure 3**). Under WLE, the macroscopic appearances of lesions were classified into five types (0-I, 0-IIa, 0-IIb, 0-IIc, and 0-III) according to the Paris endoscopic classification (20). According to the Union for International Cancer Control, the longitudinal locations within the esophagus were divided into three divisions: upper, middle, and lower (21). Dramatic color change after iodine staining (initially whitish-yellow and then pink 2 to 3 min later) was recognized as PCS (22), and the lesions were classified as PCS positive and PCS negative.

Pathological diagnosis was the gold standard for the diagnostic performance of LCE examinations for esophageal neoplastic lesions.

### Sample Size Estimation and Statistical Analysis

We assumed the estimated rate of misdiagnosis to be 37% according to the estimation in the previous study and events per variable (EPV) to be 15 (23, 24). Thirteen variables were included in the logistic regression analysis. Thus, the minimum sample size was 527 based on these assumptions and variables. In this study, a sample size of 627 lesions was sufficient for subsequent multivariate logistic analysis. The sensitivity, specificity, positive predictive value (PPV), negative predictive value (NPV), and accuracy of LCE in diagnosing esophageal neoplasms relative to the histopathologic diagnosis were calculated using McNemar’s test. Continuous variables were compared using the Mann–Whitney *U* test, and categorical variables were compared using the chi-squared test or Fisher’s exact test as appropriate. Univariate risk factors with a significance level of <0.05 were entered into a multivariate logistic regression analysis. A *p*-value of <0.05 was considered to indicate statistical significance in multivariate logistic regression analysis. Multivariate logistic regression analysis was performed to determine significant factors affecting the misdiagnosis of esophageal neoplastic lesions by LCE. Odds ratios (ORs) and 95% confidence intervals (CIs) were calculated. Statistical analysis was performed using SPSS (SPSS for Windows, version 22.0, SPSS Inc, Chicago, Ill).

## Results

### The General Characteristics of Patients and Lesions

Seven hundred and seventy-three consecutive patients with 871 lesions were investigated in this analysis. The characteristics of the patients and lesions are summarized in **Supplementary Table 1**. The male-to-female ratio was 1.4:1 (446 males: 327 females), and the median age was 57 years (range, 23–86 years). A total of 53.9% of the lesions (469/871) were located in the middle esophagus, and 38.5% (336/871) were located in the lower esophagus. A total of 76.4% (665/871) of the lesions had a branching vascular network, and 68.9% (600/871) of the lesions were hyperemic. With regard to the size of lesions, 30.8% were <5 mm, 28.4% were between 5 and 10 mm, 26.5% were between 11 and 30 mm, and 14.3% were >30 mm. A total of 47.8% (416/871) of the lesions were well demarcated, and 69.2% (603/871) of the lesions were rough. In total, 32.4% (282/871) of the lesions had PCS.

### The Diagnostic Accuracy of LCE for the Diagnosis of Esophageal Neoplastic Lesions

The biopsy results displayed based on LCE results are shown in **Table 1**. LCE-positive lesions consisted of 269 neoplastic and 358 nonneoplastic lesions, whereas all LCE-negative lesions were nonneoplastic. The sensitivity, specificity, PPV, NPV, and overall accuracy of LCE for the detection of esophageal neoplastic lesions were 100.0 (95% confidence interval, 98.2–100), 40.5 (95% confidence interval, 36.6–44.6), 42.9 (95% confidence interval, 39.0–46.9), 100.0 (95% confidence interval, 98.1–100.0), and 58.9 (95% confidence interval, 55.6–62.1), respectively (**Table 2**). The specificity of LCE was not satisfactory in diagnosing esophageal neoplastic lesions.

**Table 1:** Biopsy results displayed based on LCE results (number of lesions).

	Neoplastic*	Non-neoplastic	Total
LCE positive	269	358	627
LCE negative	0	244	244
Total	269	602	871

LCE, Lugol chromoendoscopy.

*Includes low-grade intraepithelial neoplasia (LGIN), high-grade intraepithelial neoplasia (HGIN), and invasive/advanced carcinoma.

**Table 2:** Diagnostic performance of LCE for esophageal neoplastic lesions.

Diagnostic performance	Value
Sensitivity*(95% CI) (%)	100.0 (98.2–100)
Specificity*(95% CI) (%)	40.5 (36.6–44.6)
PPV (95% CI) (%)	42.9 (39.0–46.9)
NPV (95% CI) (%)	100.0 (98.1–100.0)
Accuracy*(95% CI) (%)	58.9 (55.6–62.1)

LCE, Lugol chromoendoscopy.

*McNemar’s test was used to assess sensitivity, specificity, and accuracy.

### Multivariate Analysis of the Characteristics of Lesions Misdiagnosed as Neoplastic by LCE

Univariate analysis with 13 variates was first performed, and the results showed that the number of lesions, location, vascular network, hyperemia, plaque, size, margin, rugosity, and PCS were significant covariates (*p* < 0.05) (**Supplementary Table 2**). Morphology, erosion, nodules, and speckled esophagus were not significant covariates (*p* > 0.05). Multivariate analysis demonstrated that lesions with branching vascular network (OR 4.53, 95% CI 2.23–9.21, *p* < 0.001) and smooth appearance (OR 2.40, 95% CI 1.38–4.18, *p* = 0.002) under WLE, lesions with a size < 5 mm (OR 3.06, 95% CI 1.38–6.78, *p* = 0.006), ill-demarcated (OR 7.83, 95% CI 4.59–13.37, *p* < 0.001), and PCS-negative (OR 4.04, 95% CI 2.38-6.84, *p* < 0.001) lesions after reaction with iodine solution were independent risk factors for unstained and understained lesions misdiagnosed as neoplastic by LCE (**Table 3**).

**Table 3:** Multivariate logistic regression analysis of risk factors for misdiagnosis by LCE.

	No. of lesions	No. of misdiagnosed lesions	OR	95% CI	*p*-value
**Size (mm)**					
<5	193	156	3.06	1.38–6.78	0.006
5–10	178	88	0.91	0.43–1.95	0.808
11–30	166	73	2.21	0.97–5.01	0.059
>30	90	41	1	Reference	
**Vascular Network**					
Branching vascular network	479	312	4.53	2.23–9.21	<0.001
Disappeared vascular network	148	46	1	Reference	
**Rugosity**					
Smooth	193	151	2.40	1.38–4.18	0.002
Rough	434	207	1	Reference	
Margin*					
Ill-demarcated	327	266	7.83	4.59–13.37	<0.001
Well-demarcated	300	92	1	Reference	
**PCS**					
PCS negative	295	318	4.04	2.38–6.84	<0.001
PCS positive	424	40	1	Reference	

LCE, Lugol chromoendoscopy; PCS, Pink color sign.

*The margin of lesions under Lugol chromoendoscopy.

## Discussion

LCE is very valuable in ESCC screening due to its low cost, convenience, and low professional requirements for doctors. As with other studies, our study also found that LCE has high sensitivity but low specificity. Lesions that had a branching vascular network or smooth appearance under WLE and were size < 5 mm, ill demarcated, or PCS negative after reaction with iodine solution were more likely to be misdiagnosed. This study is the first to perform a multivariate logistic analysis on the causes of misdiagnosis in LCE, and this information can help improve the specificity of LCE and has significant clinical value for ESCC screening.

To date, the most common methods for ESCC screening include NBI and LCE. NBI can enhance the superficial and epithelial microvascular structure, which helps identify esophageal SCC at an early stage (25, 26). However, expensive devices and experienced endoscopists are required to conduct NBI endoscopy. Many patients in undeveloped and rural areas probably go to primary-level hospitals for procedures to detect ESCC. At present, however, many primary-level hospitals do not have NBI devices, and time and financial support are required to popularize the devices. In addition, it has been reported that the diagnostic accuracy of NBI in experienced endoscopists was 11%–15% higher than that of inexperienced endoscopists (11). Doctors who are sufficiently experienced to perform NBI examinations in primary-level hospitals still represent an urgent need.

However, image enhancement with chromoendoscopy using dyes has been a cost-effective option for many years. This stain is particularly useful in identifying early esophageal squamous carcinomas, which, in contrast to normal squamous epithelium, appear unstained or understained in color (27–29). More importantly, LCE is comparatively easier to master, even for trainees, and no special devices are required. Thus, LCE is highly suitable for ESCC screening, especially in primary-level hospitals. From this perspective, LCE is easily applied with a modicum of experience and will have a comparatively rapid learning curve. The current study showed that LCE has high sensitivity and low specificity, which results in a high misdiagnosis rate. Normal squamous epithelial cells are rich in glycogen, which can turn dark brown upon exposure to iodine. In some cases, however, the glycogen content in esophageal nonneoplastic lesions (esophagitis, etc.) is reduced, showing varying degrees of understained or unstained areas (30). Previous studies have reported that the specificity of LCE is generally between 40% and 90% (13). These results are consistent with the specificity obtained in this study; our result is relatively low but still within this range. Accordingly, when LCE serves as the preferred diagnostic endoscopy of screening ESCC, it is meaningful to enhance the specificity of LCE.

In this study, we analyzed the multivariant risk factors for misdiagnosis for the first time. Our study showed that “unstained” or “understained” lesions that had branching vascular networks or smooth appearances under WLE as well as lesions < 5 mm in size that were ill demarcated or PCS negative after spraying iodine were more likely to be misdiagnosed as tumorous lesions even if they were benign. In other studies, Lugol-voiding lesions (LVLs) > 5 mm in diameter were often chosen as the threshold for pathological examination, which is an empirical conclusion that lacks specific research to support it (13, 14, 31). Similarly, our study indicated that “unstained” or “understained” lesions less than 5 mm in size tended to be accompanied by a higher risk of misdiagnosis of neoplasia by LCE, which can also serve as a reference. In fact, normal mucosa of the esophagus often appears as a clear branching capillary vascular network. The vessels are radial in the upper esophagus. However, the vessels in the middle section are branch-like, and palisade vessels are present at the distal end of the esophagus (32). Of note, the branching vascular network may disappear or be disrupted in the process of esophageal neoplasia. When the branching vascular network is clearly visible, the lesion is more likely to be nonneoplastic. In addition, our research also demonstrated that if the lesions appear ill demarcated after exposure to iodine, they are more likely to be misdiagnosed as neoplasia. The reason may be that the glycogen content of neoplastic lesions is significantly lower than that of normal tissues. The transition from the adjacent normal squamous epithelium to the lesion was very abrupt in neoplasia, which is compatible with the well-demarcated margin demonstrated by LCE (18). Therefore, a clear boundary may be observed between neoplastic lesions and normal mucosa in the surroundings when it encounters iodine solution. In contrast, after exposure to iodine, the color change of the boundary among nonneoplastic lesions could be blurred. In other words, ill-demarcated lesions are less likely to be tumors. The earliest change in ESCC is that the esophageal mucosa loses its usual luster and becomes rough. These features are accompanied by changes in color, structure, and texture. In some cases, although smooth lesions are not stained with iodine, they are more likely to be nonneoplastic lesions. Several previous studies have examined the underlying mechanism of PCS (22). The PCS may form due to disruption of the normal epithelial structure and early leakage of iodine, indicating the appearance of neoplastic lesions (33). The current study has demonstrated that PCS-negative lesions are more likely to be nonneoplastic, which is consistent with previous research. In summary, unstained or understained lesions with branching vascular networks or smooth appearances under WLE, size < 5 mm in diameter, ill-demarcated, or PCS-negative lesions after exposure to iodine are more likely to be benign.

Improving the specificity of LCE will result in the correct identification of more non-neoplastic lesions that were “unstained” or “understained” before making a final diagnosis as neoplastic lesions and reduce the false-positive rate. This study established a multivariate logistic model to preliminarily predict the probability of misdiagnosis in esophageal lesions by LCE (**Supplementary Table 3**). Subsequently, the predictive ability of the logistic model was evaluated in terms of the area under the curve (AUC) in a receiver operating characteristic (ROC) curve along with 95% CIs. The AUC was calculated for the five-predictor variable logistic model based on five characteristics as predictors and misdiagnosis as a response. The estimated AUC for the five predictors was 0.826 (95% CI, 0.788–0.864, *p* < 0.05), indicating excellent performance in predicting misdiagnosis outcomes (**Supplementary Figure 1**). The main cutoff values for the ROC curve are shown in **Supplementary Table 4**. According to the Youden index, the most suitable cutoff value for misdiagnosis was 0.596 with 72.7% sensitivity and 80.6% specificity, which helped effectively identify high-risk misdiagnosed lesions and improve the predictive accuracy of the logistical model (34). During clinical endoscopy procedures, combined with endoscopists’ experience, the logistic model quantifies various characteristics and provides an objective and scientific method for predicting the misdiagnosis of neoplastic esophageal lesions. Therefore, this study holds high clinical value for ESCC screening.

The correct understanding of the false-positive rate and the improvement of specificity is of a specific reference value for biopsy. Our study is a double-center study with a large sample size, and it is the first study to discuss the multivariate risk factors for misdiagnosis under LCE. However, the main limitation of this study is its retrospective nature, and further prospective studies are needed to validate our results. However, these results are essential to develop the next steps used to conduct further studies.

In conclusion, the specificity of LCE is not satisfactory for the diagnosis of esophageal neoplastic lesions. For unstained or understained lesions, branching vascular network or smooth appearance under WLE, size < 5 mm in diameter, ill-demarcated, or PCS negative results after staining are related to the misdiagnosis of esophageal neoplastic lesions by LCE based on logistic regression. The multivariate logistic model may be used to predict the possibility of misdiagnosis and help improve the specificity of LCE in diagnosing esophageal neoplastic lesions.

## Data Availability Statement

The raw data supporting the conclusions of this article will be made available by the authors, without undue reservation.

## Ethics Statement

Written informed consent was obtained from patients before all examinations. The protocol for this study was undertaken in conformity with the provisions of the Declaration of Helsinki.

## Author Contributions

RL conceived and designed the project. ZG wrote the manuscript. ZG and LM collected, analyzed, and interpreted data. LM and ST performed imaging analysis. HS and MF performed the statistical analysis. ZG and LM contributed equally to this work. All authors contributed to the article and approved the submitted version.

## Funding

This study was supported in part by the National Natural Science Foundation of China (Nos. 81770539 and 81974068), the National Program on Key Basic Research Project of China (No. 2017YFC0110003), and the Natural Science Foundation of Hubei Province of China for Distinguished Young Scholar (No. 2017CFA061).

## Conflict of Interest

The authors declare that the research was conducted in the absence of any commercial or financial relationships that could be construed as a potential conflict of interest.

## Publisher’s Note

All claims expressed in this article are solely those of the authors and do not necessarily represent those of their affiliated organizations, or those of the publisher, the editors and the reviewers. Any product that may be evaluated in this article, or claim that may be made by its manufacturer, is not guaranteed or endorsed by the publisher.

## Abbreviations

ESCC, Oesophageal squamous cell cancer; WLI, White light imaging; LCE, Lugol chromoendoscopy; NBI, Narrow band imaging; PCS, Pink colour sign; PPV, Positive predictive value; NPV, Negative predictive value; ORs, Odds ratios; CIs, Confidence intervals; LVL, Lugol-voiding lesions; ROC, Receiver operating characteristics; AUC, Area under the curve.
