# Supplementary material for: A Five-Parameter Logistic Model to Predict the Possibility of Misdiagnosis for Improving the Specificity of Lugol Chromoendoscopy in the Diagnosis of Esophageal Neoplastic Lesions
Source: Front Oncol. 2022 Jan 3;11:763375. doi: 10.3389/fonc.2021.763375 (PMC8763246; doi:10.3389/fonc.2021.763375)
Supplement: Supplementary file 1 [file DataSheet_1.docx]

## Supplementary Tables and Figures

Supplementary Table 1. Clinical and pathological characteristics (773 patients with 871 lesions)

| **Characteristics** | **n (%)** |
| --- | --- |
| **Age, mean (range)** | 57 (23-86) |
| **Sex**  Male  Female | 446  327 |
| **Morphology,** **n (%)** |  |
| 0-I | 74 (8.5) |
| 0-IIa | 182 (20.9) |
| 0-IIb | 510 (58.5) |
| 0-IIc | 75 (8.6) |
| 0-III | 30 (3.5) |
| **Number of lesions,** **n (%)** |  |
| Multiple | 168 (19.3) |
| Single | 703 (80.7) |
| **Location at ﻿longitudinal *, n (%)** |  |
| Upper third | 65 (7.5) |
| Middle third | 469 (53.9) |
| Lower third | 336 (38.6) |
| **Vascular network, n (%)** |  |
| Branching vascular Network | 665 (76.4) |
| Disappeared vascular Network | 206 (23.6) |
| **Hyperaemia, n (%)** |  |
| Nonhyperaemia | 271 (31.1) |
| Hyperaemia | 600 (68.9) |
| **Size(mm), n (%)** |  |
| <5 | 268 (30.8) |
| 5-10 | 247 (28.4) |
| 11-30 | 231 (26.5) |
| >30 | 125(14.3) |
| **Margin^†^,** **n (%)** |  |
| Well-demarcated | 416 (47.8) |
| Ill-demarcated | 455 (52.2) |
| **Rugosity (%)** |  |
| Rough | 603 (69.2) |
| Smooth | 268 (30.8) |
| **PCS (%)** |  |
| PCS positive | 282 (32.4) |
| PCS negative | 589 (67.6) |
| **Iodine staining (%)** |  |
| Unstained | 410 (47.1) |
| Understained | 217 (24.9) |
| Normal greenish brown staining | 149 (17.1) |
| hyperstaining | 95 (10.9) |
| **Pathological diagnosis (%)** |  |
| Nonneoplasia | 497 (57.1) |
| LGIN | 187 (21.5) |
| HGIN | 103 (11.8) |
| SCC | 84 (9.6) |
| **Other characteristics, n (%)** |  |
| Erosion | 46 (5.28) |
| Nodule | 4 (0.46) |
| plaque | 58 (6.66) |
| Speckled esophagus**‡** | 44 (5.05) |

PCS, Pink color sign.

***** The location of each lesion was recorded according to the distance from the incisor teeth. Upper third: < 25 cm; middle third: 25 cm ~ 32 cm; lower third: > 32 cm.

**^†^** The margin of lesions under Lugol chromoendoscopy.

**‡** Multiple Lugol-unstained speckles were present throughout the esophagus.

Supplementary Table 2. Univariate analysis of risk factors for misdiagnosis of oesophageal neoplastic lesion by LCE.

|  | Group A  (n=358) | | | Group B  (n=269) | *P* value |
| --- | --- | --- | --- | --- | --- |
| **Morphology,** **n (%)** | | |  |  | 0.107 |
| 0-I | | | 31 (8.7) | 22 (8.6) |  |
| 0-IIa | | | 67 (18.6) | 64 (23.7) |  |
| 0-IIb | | | 218 (60.9) | 149 (55.9) |  |
| 0-IIc | | | 24 (6.7) | 30 (11.0) |  |
| 0-III | | | 18 (4.3) | 4 (1.6) |  |
| **Number of lesions,** **n (%)** | | |  |  | 0.001 |
| Multiple | 85 (23.7) | | | 36 (13.4) |  |
| Single | 273 (76.3) | | | 233 (86.6) |  |
| **Location*,** **n (%)** |  | | |  | 0.049 |
| Upper third | 32 (8.9) | | | 15 (5.6) |  |
| Middle third | 201 (56.1) | | | 137 (50.9) |  |
| Lower third | 125 (34.9) | | | 117 (43.5) |  |
| **Vascular network, n (%)** |  | | |  | <0.001 |
| Branching vascular Network | 312 (87.2) | | | 167 (62.1) |  |
| Disappeared vascular Network | 46 (12.8) | | | 102 (37.9) |  |
| **Hyperemia, n (%)** |  | | |  | <0.001 |
| Non- hyperemia | 140 (39.1) | | | 55 (20.4) |  |
| Hyperemia | 218 (60.9) | | | 214 (79.6) |  |
| **Erosion, n (%)** |  | | |  | 0.103 |
| Non-erosion | 337 (94.1) | | | 244 (90.7) |  |
| Erosion | 21 (5.9) | | | 25 (9.3) |  |
| **Nodule,** **n (%)** |  | | |  | 0.774 |
| Non-nodule | 356 (99.4) | | | 267 (99.3) |  |
| Nodule | 2 (0.6) | | | 2 (0.7) |  |
| **plaque, n (%)** |  | | |  | <0.001 |
| Non- plaque | 353 (98.6) | | | 216 (80.3) |  |
| plaque | 5 (1.4) | | | 53 (19.7) |  |
| **Speckled esophagus^†^,** **n (%)** |  | | |  | 0.723 |
| Non-speckled esophagus | 334 (93.3) | | | 249 (92.6) |  |
| Speckled esophagus | 24 (6.7) | | | 20 (7.4) |  |
| **Size(mm),** **n (%)** |  | | |  | <0.001 |
| <5 | 156 (43.5) | | | 37 (13.8) |  |
| 5-10 | 88 (24.6) | | | 90 (33.5) |  |
| 11-30 | 73 (20.4) | | | 93 (34.5) |  |
| >30 | 41 (11.5) | | | 49 (18.2) |  |
| **Margin‡,** **n (%)** |  | | |  | <0.001 |
| Well-demarcated | 92 (25.7) | | | 208 (77.3) |  |
| Ill-demarcated | 266 (74.3) | | | 61 (22.7) |  |
| **rugosity,** **n (%)** |  | | |  | <0.001 |
| Rough | 207 (57.8) | | | 227 (84.4) |  |
| Smooth | 151 (42.2) | | | 42 (15.6) |  |
| **PCS,** **n (%)** | |  | |  | <0.001 |
| PCS-positive | 40 (11.2) | | | 163 (60.6) |  |
| PCS-negative | 318 (88.8) | | | 106 (39.4) |  |
| Group A, the lesions misdiagnosed as neoplastic lesions by LCE but eventually pathologically diagnosed as non-neoplastic lesions; Group B, the lesions diagnosed as neoplastic lesions by LCE and finally confirmed by pathological examination  LCE, Lugol chromoendoscopy; PCS, Pink color sign.  ***** The location of each lesion was recorded according to the distance from the incisor teeth. Upper third: < 25 cm; middle third: 25 cm ~ 32 cm; lower third: > 32 cm.  **^†^** Multiple Lugol-unstained speckles were present throughout the esophagus.  **‡** The margin of lesions under Lugol chromoendoscopy. | | | | | |

Supplementary Table 3. Variables in the multivariable logistic regression model.

|  | Coefficient (B) | Standard Error | Wald X^2^ | Sig. | Exp (B) | 95% CI |
| --- | --- | --- | --- | --- | --- | --- |
| **Size*** |  |  | 18.21 | <0.001 |  |  |
| Size (1) | 1.12 | 0.41 | 7.53 | 0.006 | 3.06 | 1.38-6.78 |
| Size (2) | -0.09 | 0.39 | 0.06 | 0.808 | 0.91 | 0.43-1.95 |
| Size (3) | 0.79 | 0.42 | 3.56 | 0.059 | 2.21 | 0.97-5.01 |
| **Branching vascular network** | 1.51 | 0.36 | 17.35 | <0.001 | 4.53 | 2.23-9.21 |
| **Rugosity** (Smooth) | 0.88 | 0.28 | 9.62 | 0.002 | 2.40 | 1. 38-4.18 |
| **Margin^†^ (i**ll-demarcated) | 2.06 | 0.27 | 56.81 | <0.001 | 7.83 | 4.59-13.37 |
| **PCS negative** | 1.40 | 0.27 | 26.93 | <0.001 | 4.04 | 2.38-6.84 |

PCS, Pink color sign.

***** The size > 30 mm was used as a reference; Size (1), size < 5 mm; Size (2), size is between 5 and 10 mm; Size (3), size is between 11 and 30 mm.

**^†^** The margin of lesions under Lugol chromoendoscopy.

Supplementary Table 4. The main cut-off values for the ROC curve.

| **Cut-off value** | **Sensitivity** | **1-Specificity** |
| --- | --- | --- |
| 0.223 | 0.957 | 0.749 |
| 0.283 | 0.937 | 0.618 |
| 0.297 | 0.929 | 0.592 |
| 0.422 | 0.854 | 0.435 |
| 0.436 | 0.842 | 0.387 |
| 0.489 | 0.794 | 0.319 |
| 0.508 | 0.755 | 0.288 |
| 0.556 | 0.739 | 0.236 |
| 0.596 | 0.727 | 0.194 |
| 0.621 | 0.684 | 0.173 |
| 0.695 | 0.585 | 0.089 |
| 0.738 | 0.542 | 0.079 |
| 0.794 | 0.447 | 0.068 |
| 0.823 | 0.379 | 0.052 |
| 0.874 | 0.344 | 0.031 |
| 0.914 | 0.178 | 0.021 |
| 0.943 | 0.134 | 0.016 |

ROC: Receiver operating characteristics.


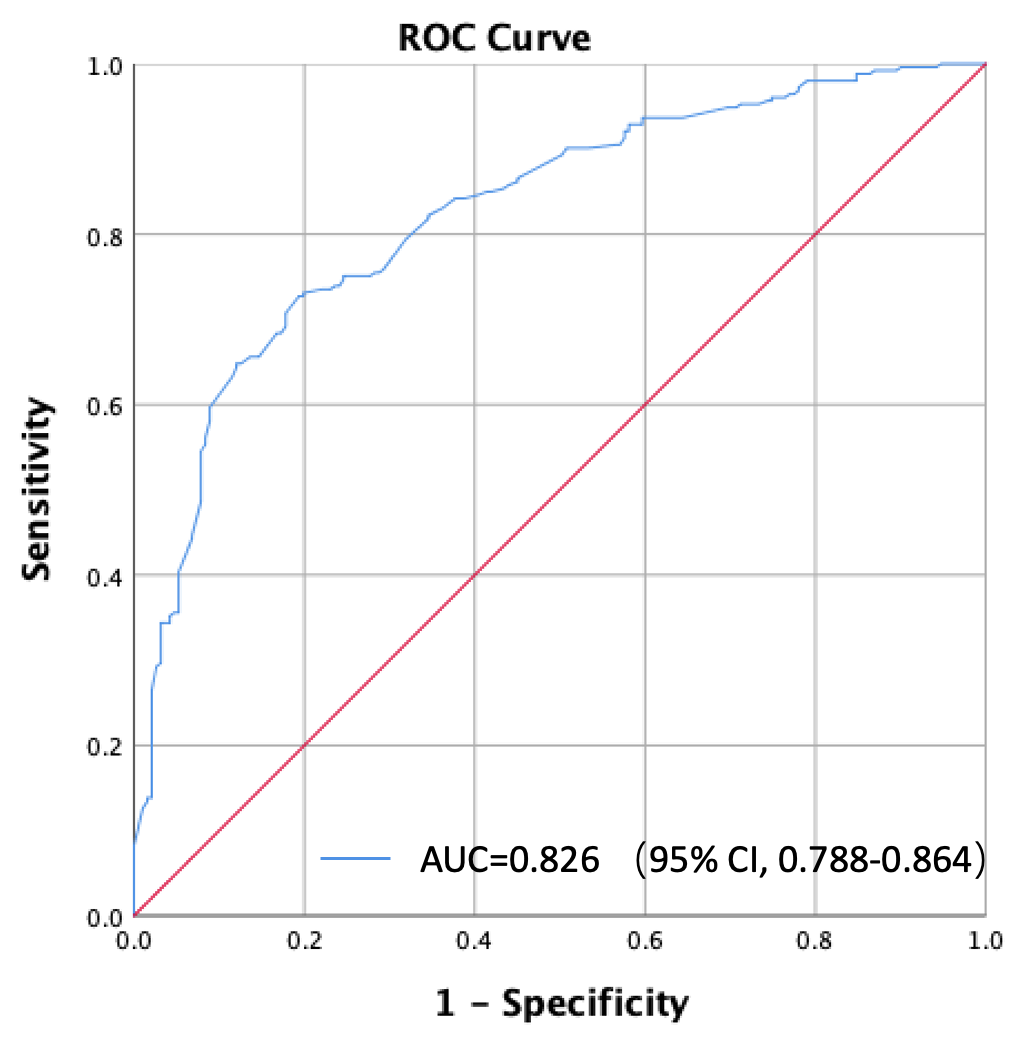
Supplementary Figure 1. Receiver operating characteristics (ROC) curve and area under the ROC curve (AUC) for assessing the possibility of misdiagnosis in oesophageal lesions by LCE.

LCE, Lugol chromoendoscopy.
